# Detecting Human-to-Human Transmission of Avian Influenza A (H5N1)

**DOI:** 10.3201/eid1312.071153

**Published:** 2007-12

**Authors:** Timothy M. Uyeki, Joseph S. Bresee

**Affiliations:** * Centers for Disease Control and Prevention, Atlanta, Georgia, USA

**Keywords:** H5N1 Virus, transmission, modeling, letter

**To the Editor:** This letter is in response to a recently published article about statistical modeling to assess human-to-human transmission of avian influenza A (H5N1) viruses in 2 case clusters (*1*). Sporadic cases and clusters of human infection with highly pathogenic avian influenza A (H5N1) viruses have occurred after direct contact with diseased or dead poultry (*2*,*3*). Limited, nonsustained human-to-human transmission of avian influenza (H5N1) viruses is believed to have occurred in some clusters (*4*). Every human infection with a novel influenza A virus should be investigated, and suspected clusters should be investigated immediately to assess exposures and transmission patterns.

Yang et al. applied a statistical model to evaluate publicly available data from 2 case clusters of human infection with avian influenza A (H5N1) viruses (*1*). These clusters were investigated in detail during 2006 by field epidemiologic investigation teams. Yang et al. suggest that statistical methods can prove or confirm human-to-human transmission, but this suggestion is misleading. Modeling approaches can suggest transmission modalities to account for case patterns, but determination of human-to-human transmission requires detailed field epidemiologic investigations in which human, animal, and environmental exposures as well as clinical and laboratory data are assessed and interpreted.

Indication that a novel influenza A virus has acquired the ability to spread among humans could be reflected by a change in the epidemiology of clusters, such as increases in 1) size and frequency of clusters, 2) cases among nonrelated persons, and 3) clinically mild cases. This ability could also be reflected in accompanying changes in viruses isolated from case-patients. When facing emerging infectious disease threats such as those posed by highly pathogenic avian influenza A (H5N1) viruses, surveillance should rapidly detect human cases and case clusters and facilitate accurate identification of the agent. Field epidemiologic investigations, initiation of evidence-based clinical management of case-patients, and epidemiologic disease-control methods (including appropriate infection control measures) should be implemented immediately. Statistical modeling can provide useful and supportive insights but should not be viewed as an alternative to a detailed field epidemiologic investigation combined with laboratory data. Timely and comprehensive field investigations remain most critical to guiding decisions about containment efforts for pandemic influenza and other emerging infectious diseases (*5*).
