# Supplementary material for: Detecting Human-to-Human Transmission of Avian Influenza A (H5N1)
Source: Emerg Infect Dis. 2007 Dec;13(12):1969–71. doi: 10.3201/eid1312.071153 (PMC2876777; doi:10.3201/eid1312.071153)
Supplement: Related texts

# Letter

**DOI:** 10.3201/EID1312.071272

**Published:** 2007-12

**Authors:** Ira M. Longini, Yang Yang, Jonathan Sugimoto, M. Elizabeth Halloran

**Affiliations:** * Fred Hutchinson Cancer Research Center, Seattle, Washington, USA; † University of Washington, Seattle, Washington, USA

## In Response

We thank Drs Uyeki and Bresee for their thoughtful commentary (*1*) on our article about assessing the possibility of human-to-human transmission of avian influenza A (H5N1) in observed clusters (*2*). We agree with them that statistical models are not substitutes for careful epidemiologic investigations combined with laboratory data. We believe that the statistical model that we advance can be an important tool to use in conjunction with sound and rapid epidemiologic investigation and intervention.

We do not claim, however, that our statistical model can prove or confirm human-to-human transmission, as Uyeki and Bresee state. In our article, we were careful to point out that we found statistical evidence of human-to-human transmission (p = 0.009) in Sumatra. We did not claim to have proven or confirmed human-to-human transmission on the basis of our statistical analysis. A strict interpretation of what we found is that the data from the cluster in northern Sumatra provided evidence to reject the null hypothesis of no human-to-human transmission. Given this, we then weighed all the epidemiologic and laboratory information available to make a scientific judgment about the likelihood of human-to-human transmission. We are not aware of any logical system analogous to Koch’s postulates (*3*) (i.e., proving the causal link between an infectious agent and clinical disease) for actually proving that an infectious agent is transmitted from person to person. However, we believe that the statistical, epidemiologic, and laboratory evidence combined weigh heavily toward the presence of limited human-to-human transmission in the case of the Sumatra cluster.

This statement can be contrasted with our analysis of the cluster in eastern Turkey, where we did not find statistical evidence of human-to-human transmission of avian influenza A (H5N1). This analysis must be evaluated with the epidemiologic and laboratory data. We simply state that we do not have enough statistical evidence to reject the null hypothesis of no human-to-human spread. Whether human-to-human transmission occurred is a scientific judgment.

We agree with Uyeki and Bresee that statistical modeling can provide useful and supportive insights, and we hope that epidemiologic teams who investigate and control potential infectious disease outbreaks will carry the TranStat software (*2*) into the field with them. We are grateful that Uyeki and Bresee point out that immediate implementation of containment measures is critical, even while we sort out the cause of an observed cluster of cases. Actually containing possible spread of a potential pandemic strain of influenza (*4**,**5*) is more important than scientific arguments about the transmissibility of the infectious agent in question.

## References

[1] Uyeki TM, Bresee JS. Detecting human-to-human transmission of avian influenza A (H5N1) [letter]. Emerg Infect Dis. 2007;13:1969–70.1825806810.3201/eid1312.071153PMC2876777

[2] Yang Y, Halloran ME, Sugimoto J, Longini IM Jr. Detecting human-to-human transmission of avian influenza A (H5N1). Emerg Infect Dis. 2007;13:1348–53.1825210610.3201/eid1309.07-0111PMC2857285

[3] Evans AS. Causation and disease: the Henle-Koch postulates revisited. Yale J Biol Med. 1976;49:175–95.782050PMC2595276

[4] Longini IM, Nizam A, Xu S, Ungchusak K, Hanshaoworakul W, Cummings DA, Containing pandemic influenza at the source. Science. 2005;309:1083–7. 10.1126/science.111571716079251

[5] Yang Y, Longini IM, Halloran ME. A resampling-based test to detect person-to-person transmission of infectious disease. The Annals of Applied Statistics. 2007;1:211–28. 10.1214/07-AOAS10519436773PMC2680309
